# Helios expression coordinates the development of a subset of striatopallidal medium spiny neurons

**DOI:** 10.1242/dev.138248

**Published:** 2017-04-15

**Authors:** Raquel Martín-Ibáñez, Mónica Pardo, Albert Giralt, Andrés Miguez, Inés Guardia, Lucile Marion-Poll, Cristina Herranz, Miriam Esgleas, Gerardo Garcia-Díaz Barriga, Michael J. Edel, Carlos Vicario-Abejón, Jordi Alberch, Jean-Antoine Girault, Susan Chan, Philippe Kastner, Josep M. Canals

**Affiliations:** 1 Stem Cells and Regenerative Medicine Laboratory, Production and Validation Center of Advanced Therapies (Creatio), Department of Biomedical Sciences, Faculty of Medicine and Health Sciences, University of Barcelona, 08036 Barcelona, Spain; 2 Neuroscience Institute, University of Barcelona, 08036 Barcelona, Spain; 3 August Pi i Sunyer Biomedical Research Institute (IDIBAPS), 08036 Barcelona, Spain; 4 Networked Biomedical Research Centre for Neurodegenerative Disorders (CIBERNED), Spain; 5 Research and Development Unit, Production and Validation Center of Advanced Therapies (Creatio), Faculty of Medicine and Health Sciences, University of Barcelona, 08036 Barcelona, Spain; 6 Pathophysiology of Neurodegenerative Diseases Laboratory, Production and Validation Center of Advanced Therapies (Creatio), Department of Biomedical Sciences, Faculty of Medicine and Health Sciences, University of Barcelona, 08036 Barcelona, Spain; 7 Inserm UMR-S839; Université Pierre et Marie Curie (UPMC, Paris 6), Sorbonne Universités; Institut du Fer à Moulin, 75005 Paris, France; 8 Control of Pluripotency Laboratory, Department of Biomedical Sciences, Faculty of Medicine and Health Science, University of Barcelona, 08036 Barcelona, Spain; 9 Victor Chang Cardiac Research Institute, Sydney, New South Wales, 2010 Australia; 10 School of Medicine and Pharmacology, Anatomy, Physiology and Human Biology, CCTRM, University of Western Australia, Western Australia, 6009 Australia; 11 Departamento de Neurobiología Molecular, Celular y del Desarrollo, Instituto Cajal, Consejo Superior de Investigaciones Científicas (CSIC), 28002 Madrid, Spain; 12 Department of Functional Genomics and Cancer, Institut de Génétique et de Biologie Moléculaire et Cellulaire (IGBMC), Inserm U964, Centre National de la Recherche Scientifique (CNRS) UMR 7104, 67400 Illkirch-Graffenstaden, France; 13 Faculté de Médecine, Université de Strasbourg, 67081 Strasbourg, France

**Keywords:** Ikaros, Neurogenesis, Medium spiny neurons, Cell cycle, Cell death, Ikzf2

## Abstract

Here, we unravel the mechanism of action of the Ikaros family zinc finger protein Helios (He) during the development of striatal medium spiny neurons (MSNs). He regulates the second wave of striatal neurogenesis involved in the generation of striatopallidal neurons, which express dopamine 2 receptor and enkephalin. To exert this effect, He is expressed in neural progenitor cells (NPCs) keeping them in the G_1_/G_0_ phase of the cell cycle. Thus, a lack of He results in an increase of S-phase entry and S-phase length of NPCs, which in turn impairs striatal neurogenesis and produces an accumulation of the number of cycling NPCs in the germinal zone (GZ), which end up dying at postnatal stages. Therefore, *He^−/−^* mice show a reduction in the number of dorso-medial striatal MSNs in the adult that produces deficits in motor skills acquisition. In addition, overexpression of *He* in NPCs induces misexpression of DARPP-32 when transplanted in mouse striatum. These findings demonstrate that He is involved in the correct development of a subset of striatopallidal MSNs and reveal new cellular mechanisms for neuronal development.

## Abstract (precis)

**Summary:** The transcription factor Helios regulates G1-S transition to promote neuronal differentiation of a striatopallidal neuronal subpopulation involved in motor skill acquisition.

## INTRODUCTION

The mammalian striatum controls body movements through a sophisticated neuronal network that is dependent on the neurogenesis of two major classes of striatal neurons: the striatal projection neurons (or medium spiny neurons; MSNs) and the interneurons. MSNs are subdivided into two subpopulations: neurons that constitute the direct (or striatonigral) pathway and preferentially express substance P (SP) and D1R (dopamine receptor 1; DRD1), and neurons of the indirect (or striatopallidal) pathway, which mainly express enkephalin (ENK) and D2R (dopamine receptor 2; DRD2) (Gerfen, 1992). These two populations are differentially distributed within the striatal compartments. Striatal patches or striosomes mainly contain SP^+^ MSNs, but both MSN subpopulations, SP^+^ and ENK^+^, are located in the matrix (Gerfen, 1992).

During embryonic development, radial glial cells (RGCs) from the ventricle wall of the lateral ganglionic eminence (LGE) undergo successive divisions to expand the pool of neural progenitor cells (NPCs), thereby increasing the volume of the germinal zone (subventricular zone; SVZ) (for reviews, see Götz and Barde, 2005; Merkle and Alvarez-Buylla, 2006). At certain developmental stages, NPCs differentiate into immature neurons that migrate radially to the mantle zone (MZ) (Götz and Barde, 2005; Merkle and Alvarez-Buylla, 2006; Mérot et al., 2009). Two waves of striatal neurogenesis segregate MSNs into two principal compartments: the patches, generated during the first neurogenic wave [starting at embryonic day (E) 12.5 in mouse]; and the matrix, developed during late striatal neurogenesis (starting at E14.5 in mouse) (Gerfen, 1992; Mason et al., 2005).

Within the LGE, transcription factors such as Gsx1 and Gsx2 (formerly named Gsh1 and Gsh2), Ascl1 (formerly named Mash1) and members of the Dlx family display specific patterns of expression within the GZ and the MZ, and they have been implicated in LGE patterning and/or differentiation (Eisenstat et al., 1999; Rallu et al., 2002; Waclaw et al., 2009; Yun et al., 2002). In addition, the transcription factors Ebf1, Isl1, Ctip2 (also known as Bcl11b), and Ikaros family members are mainly expressed in the MZ of the LGE where they regulate terminal differentiation of striatal projection neurons (Arlotta et al., 2008; Ehrman et al., 2013; Garcia-Dominguez et al., 2003; Garel et al., 1999; Lobo et al., 2006, 2008; Martín-Ibáñez et al., 2010).

Ikaros family members are transcription factors that play essential roles during lymphocyte development (Cobb and Smale, 2005; Georgopoulos, 2002; Yoshida and Georgopoulos, 2014). Ikaros is the founder member of this family of DNA-binding proteins, which consists of Ikaros, Helios (He), Aiolos, Eos and Pegasus (Ikzf1-5, respectively – Mouse Genome Informatics) (John et al., 2009; Rebollo and Schmitt, 2003; Yoshida and Georgopoulos, 2014). In addition, Ikaros has been implicated in CNS development (Agoston et al., 2007; Alsiö et al., 2013; Martín-Ibáñez et al., 2010). We have recently described that He is also implicated in striatal development (Martín-Ibáñez et al., 2012). Within the LGE, *He* is expressed from E14.5 to postnatal day (P) 15 in both the GZ and the MZ, and its expression is downstream of *Gsx2* and *Dlx1/2* (Martín-Ibáñez et al., 2012). However, little is known about mechanisms of action of He during this developmental process.

Here, we demonstrate that *He* is expressed by NPCs at the G_0_/G_1_-phase of the cell cycle and induces neuronal differentiation by decreasing the levels of cyclin E and blocking the progression of these NPCs into S phase. Consequently, in the absence of *He*, proliferating NPCs accumulate in the GZ and the number of *Ctip2*^+^ and DARPP-32 (PPP1R1B)^+^ MSNs is reduced in the striatum resulting in disturbance of motor skill learning.

## RESULTS

### *He* loss induces aberrant striatal neurogenesis accompanied by de-regulation of NPC proliferation

**Fig. 1.**
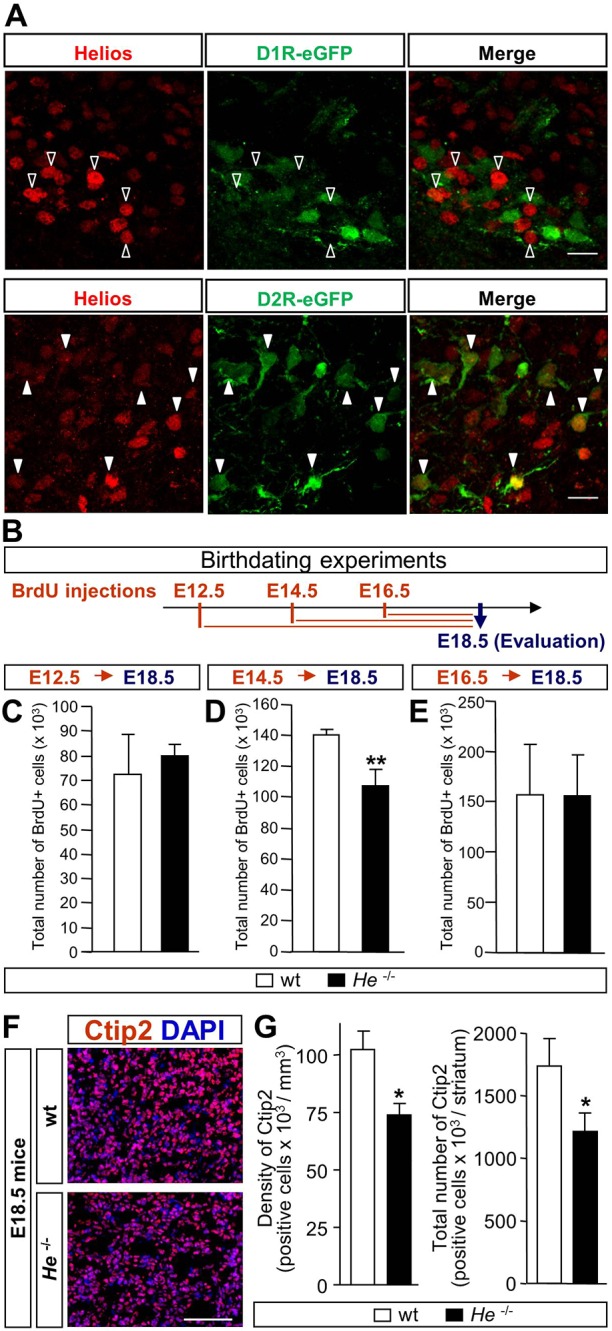
***He* is necessary for the second wave of striatal neurogenesis.** (A) Double immunohistochemistry against He and GFP in the D1R-eGFP mice and in the D2R-eGFP mice (images show DLS and VLS, respectively). Unfilled arrowheads show single-labeled cells and filled arrowheads show double-positive cells. Scale bars: 15 μm. (B) Schematic timeline of birthdating experiments performed in *He*^−/−^ or wt mice. (C) No differences in neurogenesis were detected at E12.5 between *He*^−/−^ and wt mice. (D) *He*^−/−^ mice exhibited lower levels of neurogenesis than wt mice at E14.5. (E) No differences in neurogenesis were detected at E16.5 between *He*^−/−^ and wt mice. (F) Representative images of Ctip2^+^ neurons in the E18.5 (mid-striatal primordium is shown). Scale bar: 120 µm. (G) Quantification of the density and total number of Ctip2*^+^* cells in the whole striatal primordium reveals a significant reduction in *He*^−/−^ mice compare with wt mice. Results represent the mean±s.e.m. of 4-5 mice per condition. Statistical analysis was performed using Student's *t*-test; *P<0.05, **P<0.005.

Here, we demonstrated that He is expressed from E12.5 in scattered cells (Fig. S1) until P15 peaking at E18.5 (Martín-Ibáñez et al., 2012). He showed preferential expression in D2R-eGFP neurons (mean±s.e.m.: 46.69±8.37% of He^+^ cells co-labeled with D2R; Fig. 1A; Fig. S2B) and *Penk* (preproenkephalin)^+^ MSNs (89.05±5.77% of He^+^ cells co-labeled with *Penk*; Fig. S3). In contrast, few D1R-eGFP^+^ neurons and *Tac1* (tachykinin A, also known as tachykinin 1)^+^ neurons co-expressed He (3.94±2.53% and 18.20±2.1% of He^+^ cells co-labeled with D1R and *Tac1*, respectively; Fig. 1A; Fig. S2A; Fig. S3B,C). We next examined striatal birth-dating in *He* knockout (*He*^−/−^) and wild-type (wt) mice at different embryonic developmental stages (Fig. 1B-E). The first wave of striatal birthdating at E12.5 was not altered, as no differences were found in the total number of bromodeoxyuridine (BrdU)^+^ cells between *He*^−/−^ and wt mice (Fig. 1C). However, lack of *He* induced a significant reduction in the second wave of striatal birthdating at E14.5 (Fig. 1D). No significant differences were found between genotypes at E16.5 (Fig. 1E). This striatal birthdating impairment disturbed MSN generation as the density and total number of Ctip2-positive cells was decreased in *He*^−/−^ mice compared with wt mice at E18.5 (Fig. 1F,G), suggesting a defect in the second neurogenic wave. In agreement, we observed that He^+^ cells were mainly generated during the second wave of striatal neurogenesis (Fig. S4), between E14.5 (Figs. S4E-G) and E16.5 (Figs. S4H-J). Only a few cells were observed to be born at earlier stages (E13.5; Figs. S4B-D).

**Fig. 2.**
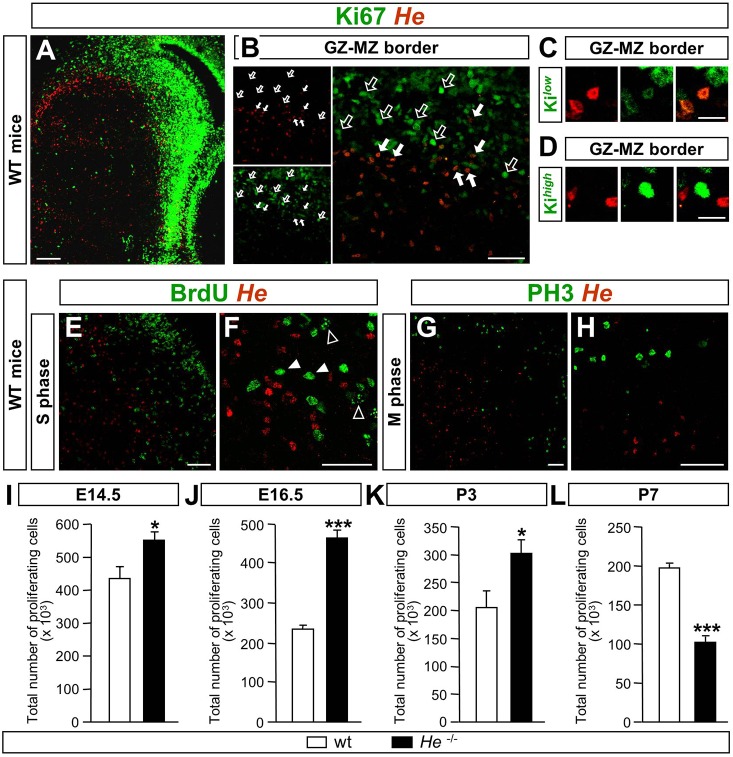
**He** **is expressed in NPCs at G_1_ cell cycle phase and regulates their proliferation.** (A) E16.5 striatal primordium, double stained against Ki67 and He. He^+^ and Ki67^+^ cells are coincident at the GZ-MZ border. Scale bar: 200 µm. (B) High magnification image of Ki67-He double immunohistochemistry at the dorsal striatal primordium shows that some cells are double positive at the GZ-MZ border. Filled arrows indicate double-positive cells and unfilled arrows point to Ki67 single-labeled cells. Scale bar: 50 µm. (C,D) At the GZ-MZ border, cells expressing a low level of Ki67 (Ki*^low^*) express He (C), whereas cells expressing a high level of Ki67 (Ki*^high^*) do not express He (D). Scale bars: 20 µm. (E,F) Double staining for BrdU and He shows that cells in S phase are not positive for He at E14.5 in the dorsomedial LGE. (E) High magnification picture shows that although He^+^ and BrdU^+^ cells are located in the same area, they do not colocalize. (F) Unfilled arrowheads indicate BrdU^+^ cells that have recently entered S phase as shown by the appearance of transcription units; filled arrowheads indicate cells that incorporated BrdU at more advanced cell cycle stages. Scale bars: 50 µm. (G,H) There is no coincidence between He-expressing cells and cells in M phase as detected by PH3 staining; low (G) and high (H) magnification images of DMS are shown. Scale bars: 50 µm. (I-L) Quantification of the total number of proliferating cells in the whole GZ show that lack of *He* induces a significant increase at E14.5 (I), E16.5 (J) and P3 (K) and a significant decrease at P7 (L) compared with wt mice. Results represent the mean±s.e.m. of 5-7 mice per condition. Statistical analysis was performed using Student's *t*-test; *P<0.05, ***P<0.001.

To assess whether He was expressed by proliferative cells in the LGE, we performed double staining for He and Ki67 (Mki67) at E16.5, BrdU or phospho-histone H3 (PH3) at E14.5. Our results showed that He^+^ and Ki67^+^ areas were mainly coincident at the GZ-MZ border at E16.5 (Fig. 2A). Within this area, He was expressed by NPCs expressing a low level of Ki67 (Fig. 2B,C) but not by cells expressing a high level of Ki67^+^ (Fig. 2D; see Fig. S5 for quantification details). However, there was a lack of colocalization between He and short-pulsed BrdU NPCs (Fig. 2E,F), and He and PH3^+^ NPCs (Fig. 2G,H). Interestingly, He only colocalized with Ki67-expressing cells during the neurogenic period as we could not observe colocalization from E18.5 onwards (Fig. S6).

Analysis of the number of cycling cells at different developmental stages in *He*^−/−^ and wt mice (Fig. 2I-L) showed that the total number of proliferating cells in the GZ was significantly increased from E14.5 to P3 (Fig. 2I-K), inducing an enlargement of the proliferative area stained with Ki67 (Fig. S7). Interestingly, this feature reverted at P7, when the number of proliferating cells in *He*^−/−^ mice decreased with respect to wt mice (Fig. 2L; Fig. S8). To analyze whether a specific subpopulation of progenitors was more compromised than others, we counted the percentage of PH3^+^ basal, subapical and apical progenitors as described by Pilz et al. (2013) (Fig. S9A,B). No differences were found between *He^−/−^* and wt mice (Fig. S9B). We also analyzed by QPCR the expression of striatal progenitor markers at E16.5. No differences were found in the levels of mRNA for these markers in *He^−/−^* compared with wt mice (Fig. S9C).

To elucidate further the role of He in NPC proliferation, we performed loss-of-function (LOF) and gain-of-function (GOF) *in vitro* studies using a neurosphere assay (Fig. S10). There was an increase in the number of proliferating cells in the absence of *He* (Fig. S10A,C,E,F). Accordingly, *He* overexpression significantly reduced the number of proliferating NPCs with respect to the control eGFP overexpressing NPCs (Fig. S10B,D). In addition, in the absence of *He*, NPCs were less prone to differentiate to β-III-tubulin^+^ neurons (Fig. S10H). In contrast, an increase in the number of neurons was observed after *He* overexpression (Fig. S10I-K). Interestingly, *He* did not exert any change in the percentage of GFAP^+^ cells in the LOF or in the GOF experiments (Fig. S10H,I). Consequently, *He^−/−^* mice did not present any defects in astrocyte differentiation compared with wt mice (Fig. S11A-D). In fact, we did not observe colocalization between He and GFAP (Fig. S11E).

### He controls proliferation through regulation of the G_1_-S checkpoint

**Fig. 3.**
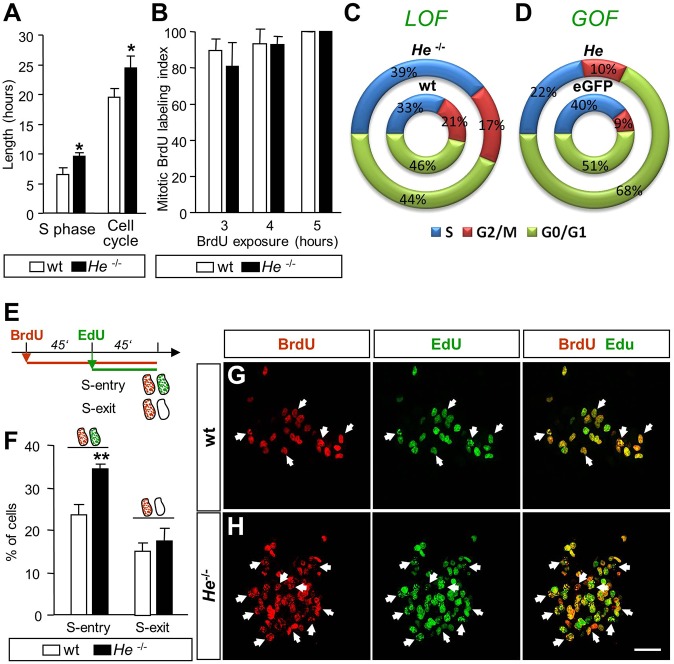
***He* is necessary for cell cycle S-phase regulation.** (A) *He*^−/−^ mice-derived neurospheres exhibited an increase in the length of S phase and cell cycle compared with wt mice-derived neurospheres. (B) Mitotic BrdU labeling index, which is used to calculate G_2_/M phase length, was the same in both wt and *He^−/−^* mice-derived neurospheres. (C,D) Schematic of the percentages of the length of the cell cycle phases with respect to the total cell cycle duration obtained from LOF (C) and GOF (D) experiments. (E) Schematic timeline of S-phase entry/exit experiments performed with a double pulse of BrdU and EdU in wt and *He*^−/−^ mice-derived neurospheres. (F) A higher number of NPCs entered S phase in *He*^−/−^ mice-derived neurospheres compared with wt mice-derived ones, whereas no differences were observed between both cultures in the number of cells that exit S phase. (G,H) Representative images of BrdU and EdU double staining performed in wt and *He*^−/−^ mice-derived neurospheres. Arrows indicate double-positive cells. Scale bar: 50 µm. Results represent the mean±s.e.m. of 4-5 LGE-derived neurosphere cultures. Statistical analysis was performed using Student's *t*-test; *P<0.05, **P<0.005.

To understand the cellular mechanism by which He regulates NPC proliferation and neurogenesis, we next analyzed the cell cycle. We observed that lack of *He* induced a significant increase in NPC S-phase length that, in turn, increased cell cycle length as measured by an accumulative exposure to BrdU (see Materials and Methods; Lange et al., 2009) (Fig. 3A,C). However, no differences were observed between the length of the G_2_/M phases in NPCs derived from *He*^−/−^ compared with wt mice, as determined by analysis of the mitotic BrdU labeling index as described previously (Takahashi et al., 1995) (Fig. 3B,C; Fig. S12). Representation of the percentage of cell cycle phases respect to the total cell cycle length clearly demonstrated an elongation of S-phase length when *He* was knocked down (Fig. 3C). Consistently, *He* overexpression induced a severe reduction of S-phase length (GOF; Fig. 3D). Our results also showed that in the absence of *He* more NPCs entered S phase (punctate BrdU^+^/EdU^+^; Fig. 3E-H) but the number of cells exiting S phase was not altered (BrdU^+^/EdU^−^; see ‘S-phase analysis’ in Materials and Methods; Lange et al., 2009) (Fig. 3E,F). In addition, no differences were found in the number of cells exiting the cell cycle (BrdU^+^/Ki67^−^; see ‘Cell cycle index’ in Materials and Methods; Urbán et al., 2010) in LOF (Fig. S13A,B,D) or GOF (Fig. S13C) experiments.

**Fig. 4.**
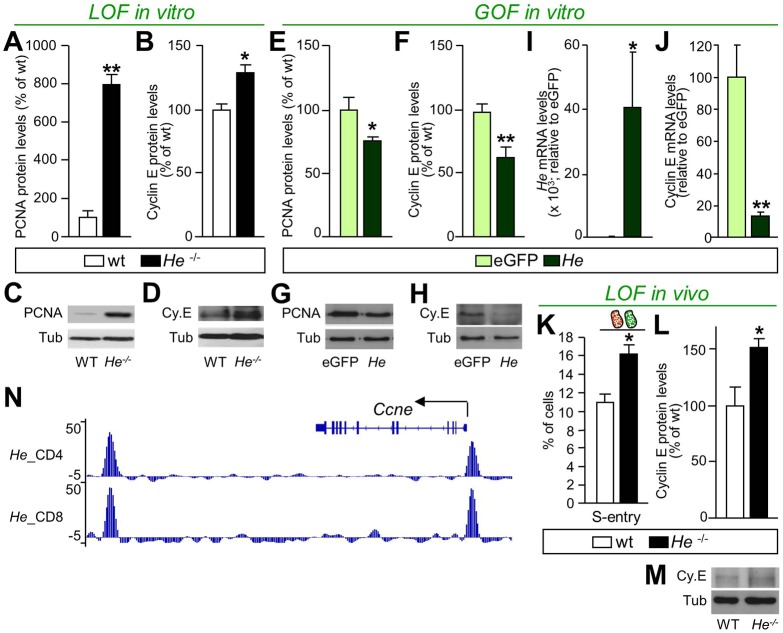
**He regulates cyclin E expression.** (A-D) PCNA (A,C) and cyclin E (Cy.E; B,D) protein quantification show a significant increase in the levels of both proteins in *He^­­−/−^*-derived neurospheres compared with wt neurospheres. Representative blots are shown for PCNA (C) and cyclin E (D). (E-H) By contrast, *He* overexpression induces a significant decrease in PCNA (E,G) and cyclin E (F,H) protein levels compared with the control eGFP. Representative blots are shown for PCNA (G) and cyclin E (H). (I) mRNA expression of *He* in neurosphere cultures overexpressing *He* or the control eGFP. (J) Cyclin E mRNA levels are downregulated in *He* overexpressing neurospheres compared with the control eGFP. (K) *In vivo* analysis shows an increased percentage of cells entering into S phase in *He^−/−^* LGEs compared with wt at E14.5. (L,M) Quantification of *He^−/−^* and wt E14.5 LGEs indicates significantly increased protein expression of cyclin E in the absence of *He*. (M) Representative blots are shown for cyclin E in LOF *in vivo* experiments. (N) Cumulative counts peak graph from the chip-Seq analysis of *He* interaction. The cyclin E (*Ccne1*) gene region shows two prominent hits one within the proximal promoter region, and one downstream of the gene. Tubulin (Tub) was used as loading control for western blots. For *in vitro* studies, results represent the mean±s.e.m. of 4-5 LGE-derived neurosphere cultures. RT-PCR results represent the mean±s.e.m. of 4-5 independent samples and are expressed relative to control eGFP, considered as 100%. For *in vivo* studies, results represent the mean±s.e.m. of 4-5 LGEs. Statistical analysis was performed using Student's *t*-test; *P<0.05, **P<0.005.

In order to demonstrate the mechanism by which He controls S-phase entry, we next analyzed the protein levels of cyclin E (Fig. 4), a key regulator of the transition from G_1_ to S phase (Ohtsubo et al., 1995). NPCs derived from *He^−/−^* mice presented increased levels of PCNA, a marker of cell proliferation, and cyclin E (Fig. 4A-D). Accordingly, *He* overexpression (Fig. 4E-H) produced a reduction of PCNA and cyclin E protein levels (Fig. 4E-H), and a drastic reduction of cyclin E mRNA levels (Fig. 4J). Similarly, *in vivo* analysis showed that an increased number of NPCs had entered into S phase in the GZ of *He^−/−^* compared with wt mice (Fig. 4K), which was accompanied by increased protein levels of cyclin E in the LGE (Fig. 4L,M). Chromatin immunoprecipitation experiments performed by Kim and co-workers (Kim et al., 2015) demonstrated that He binds the cyclin E gene (*Ccne1*) promoter site and another site downstream of the gene (Fig. 4N). However, no changes of the two cyclin E regulators E2F1 and retinoblastoma (Rb; Rb1) (Harbour, 2000; Ohtani et al., 1995) were observed in NPCs derived from *He^−/−^* mice (Fig. S14). Altogether, these results suggest that He might control cell cycle progression through regulation of cyclin E expression.

### Postnatal cell death is increased in *He^−/−^* mice

**Fig. 5.**
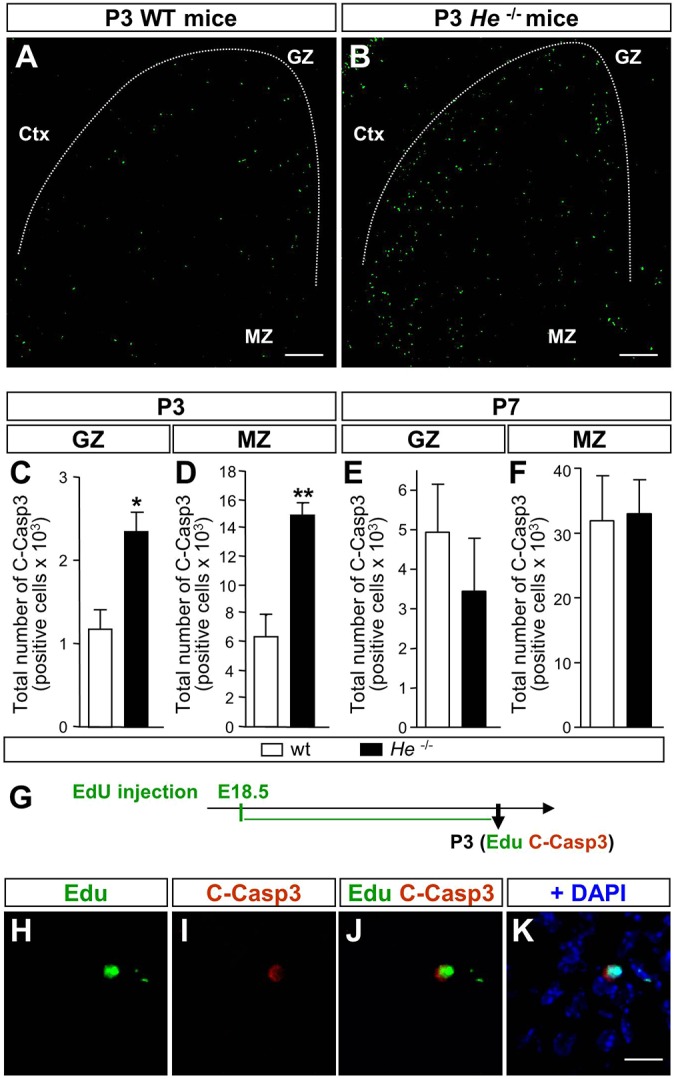
***He* knockout mice** **exhibit** **increased programmed cell death at postnatal stages.** (A,B) Representative photomicrographs corresponding to P3 striatal coronal sections from wt (A) and *He*^−/−^ (B) mice immunostained for cleaved caspase 3. Scale bars: 200 µm. Ctx, cortex. (C) Lack of *He* induces a significant increase in the total number of cleaved caspase-3 (C-Casp3)^+^ cells in the GZ at P3. (D) *He*^−/−^ mice exhibited an increase in the total number of C-Casp3^+^ cells in the MZ at P3 compared with wt mice. (E,F) No differences in the total number of C-Casp3^+^ cells were observed between genotypes in the GZ (E) or in the MZ at P7 (F). (G) Injection of EdU at E18.5 and recovery of the *He*^−/−^ pups at P3 permitted the examination of whether cells that exit the cell cycle after E18.5 and migrate to the striatum MZ are positive for C-Casp3. (H-K) Representative photomicrographs of striatal coronal ventral section showing colocalization of EdU and C-Casp3. Scale bar: 30 µm. Results represent the mean±s.e.m. of 4-5 mice per condition. Statistical analysis was performed using Student's *t*-test; *P<0.05, **P<0.005.

We next investigated whether cell death was altered in the absence of *He* during embryonic and postnatal stages. Cleaved caspase-3 immunohistochemistry did not reveal any differences between *He*^−/−^ and wt mice at embryonic stages (E14.5, E16.5 and E18.5; data not shown). However, a significant increase in the number of apoptotic cells was detected in the GZ and the MZ at P3 in *He*^−/−^ mice (Fig. 5A-D), which normalizes at P7 (Fig. 5E,F). To check whether cell death is related to a delay in the differentiation of NPCs, we applied an ethynyl deoxyuridine (EdU) pulse at E18.5 and double staining for EdU and cleaved caspase-3 (Fig. 5G) or neural markers (Fig. S15) at P3. EdU^+^ apoptotic cells were found in the MZ of *He*^−/−^ mice (Fig. 5H-K) and they were positive for the neuronal marker NeuN (Rbfox3) (71.3±7.10% of cleaved caspase-3^+^ cells co-labeled with NeuN; Fig. S15). These results suggest that in the absence of *He* there is a delayed differentiation of NPCs, which subsequently die.

### *He* is necessary for MSN development

**Fig. 6.**
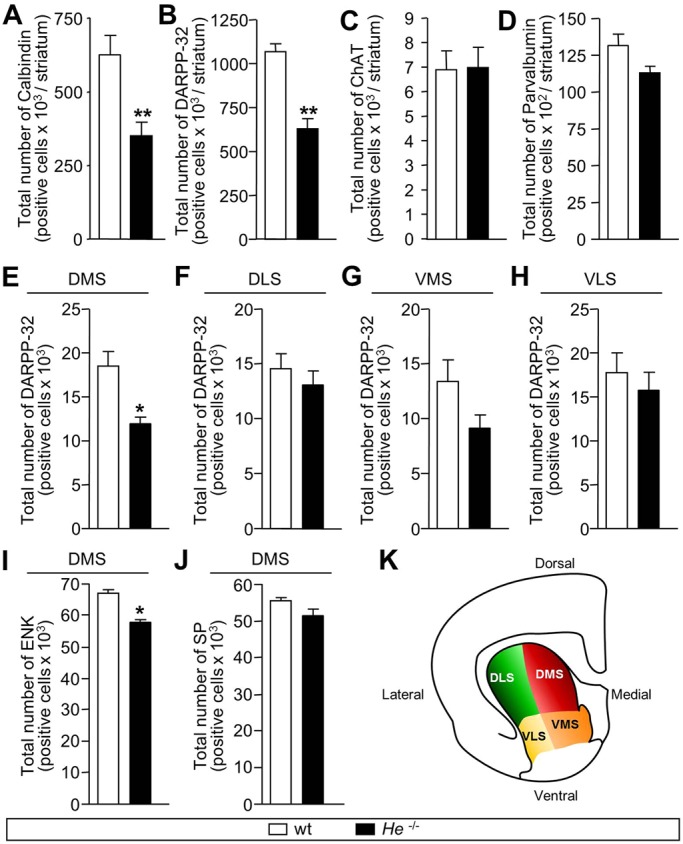
**Lack of *He* during development alters the number of mature MSNs in adult *He*^−/−^ mice.** (A-J) Stereological cell counts of neuronal striatal populations in wt and *He*^−/−^ mice striatum. (A) The total number of striatal calbindin^+^ cells is reduced in *He*^−/−^ adult mice compared with wt adult mice. (B) The total number of striatal DARPP-32^+^ cells is reduced in *He*^−/−^ adult mice compared with wt adult mice. (C,D) The total number of striatal ChAT^+^ (C) or parvalbumin^+^ (D) cells is not altered between wt and *He*^−/−^ adult mice. (E-H) The total number of striatal DARPP-32^+^ cells is specifically reduced in the DMS (E) in *He*^−/−^ adult mice compared with wt adult mice. No differences are found in the DLS (F), VMS (G) and VLS (H) between both genotypes. (I) The total number of ENK^+^ cells is reduced in the DMS of *He*^−/−^ compared with wt mice. (J) The total number of SP^+^ cells is not altered in the DMS between wt and *He*^−/−^ mice. (K) Schematic showing the division of a coronal striatal section into DMS, DLS, VMS and VLS regions. Results represent the mean±s.e.m. of 4-5 mice per condition. Statistical analysis was performed by using Student's *t*-test; *P<0.05, **P<0.005.

We next characterized the striatum of *He*^−/−^ adult mice. First, we studied brain hemisphere volume and detected a slight decrease in *He*^−/−^ mice compared with wt mice (Fig. S16A,C; 8.36% decrease). Interestingly, characterization of striatal volume revealed a larger and significant reduction in *He*^−/−^ compared with wt mice (Fig. S16B,C; 20.17% decrease). The ratio of striatal versus hemisphere volume showed that striatal volume is selectively disturbed in *He*^−/−^ mice (wt, 18.23±0.79%; *He*^−/−^, 15.45±0.60%), showing a 15.24% reduction of relative striatal volume. Stereological analysis of calbindin^+^ and DARPP-32^+^ neurons revealed a significant decrease in the density (Fig. S16D,E,H,I) and total number of MSNs in the striatum of *He*^−/−^ compared with wt mice (Fig. 6A,B). We also analyzed the density of DARPP-32^+^ neurons in different striatal areas including the dorso-medial striatum (DMS), dorso-lateral striatum (DLS), ventro-medial striatum (VMS) and ventro-lateral striatum (VLS) (Fig. 6K). These experiments demonstrated a significant decrease only in the DMS in *He*^−/−^ mice compared with wt mice (Fig. 6E-H). Interestingly, a specific alteration of the ENK^+^ population was also observed in the DMS in the absence of *He* (Fig. 6I). However, no differences were found for the SP^+^ population in *He*^−/−^ mice compared with wt mice (Fig. 6J). In addition, no differences were observed between genotypes in the cholinergic and parvalbumin^+^ striatal interneurons (Fig. S16F,G; Fig. 6C,D).

**Fig. 7.**
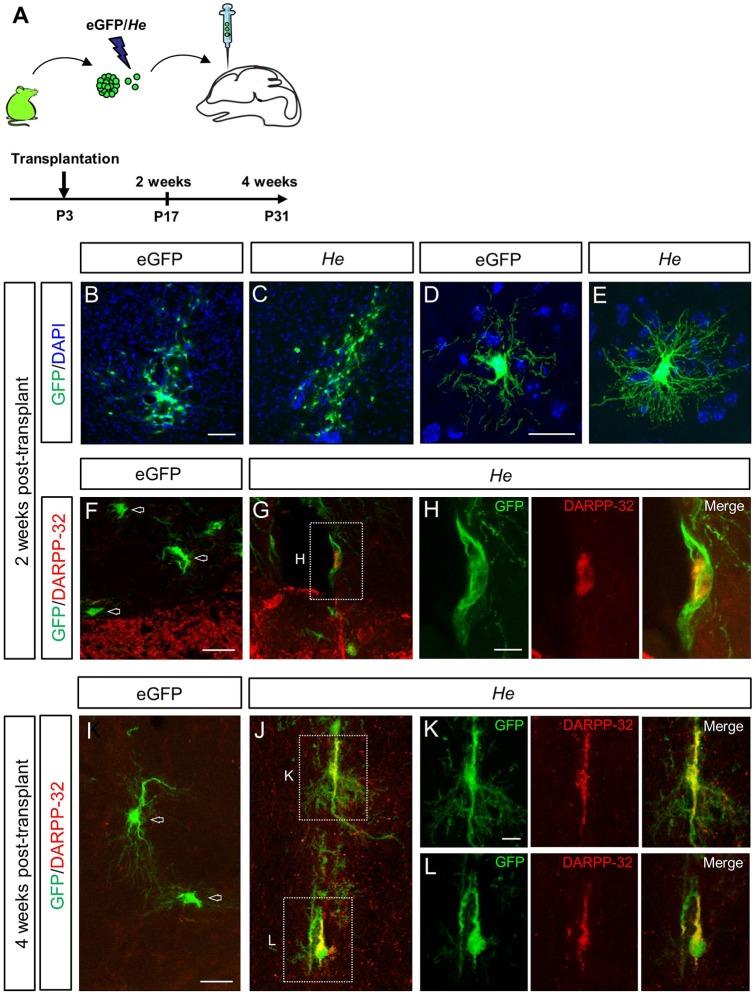
***He* induces an MSN phenotype *in vivo*.** (A) Schematic of the transplantation of eGFP and *He*-overexpressing NPCs into the mouse neonatal forebrain. (B-H) Representative images of forebrain coronal sections containing grafted cells 2 weeks post-transplantation, immunostained for GFP and DARPP-32. Compared with control cells (B,D), *He* overexpressing cells display more robust branching (C,E) and a few of them start to express DARPP-32 (G,H). (I-L) Representative images of grafted cells 4 weeks post-transplantation, labeled for GFP and DARPP-32. In contrast to control cells (I), several *He* overexpressing cells display DARPP-32 expression (J-L), indicative of the acquisition of a striatal MSN fate. Scale bars: 50 µm (B,C); 20 µm (D-G,I,J); 10 µm (H,K,L).

In order to study the direct involvement of He in the acquisition of a mature MSN phenotype, we transplanted eGFP or *He*-overexpressing NPCs into the mouse neonatal forebrain (Fig. 7A). Compared with control cells, *He*-overexpressing cells displayed more robust branching 2 weeks post-transplantation (total neurite tree length per neuron: GFP 168.13±21.92 µm, *He* 413.66±98.84 µm, *P*=0.0046; number of branches per neuron: GFP 14.43±1.68, *He* 24.89±4.08, *P*=0.0089; Fig. 7B-E) and DARPP-32 expression was observed in few scattered cells adjacent to the striatum (Fig. 7G,H). Four weeks post-transplantation, several *He*-overexpressing cells displayed DARPP-32 expression (Fig. 7J-L), in contrast to control cells, which were all DARPP-32 negative (Fig. 7I). Quantification of DARPP-32^+^ neurons in GFP transplanted cells demonstrated a 150-fold increase in the number of double-stained cells in He-expressing cells compared with controls. In addition, *He* overexpression in striatal primary cultures significantly increased the number of calbindin^+^, DARPP-32^+^ and ENK^+^ cells (Fig. S17).

### *He* loss disturbs the acquisition of motor skills

**Fig. 8.**
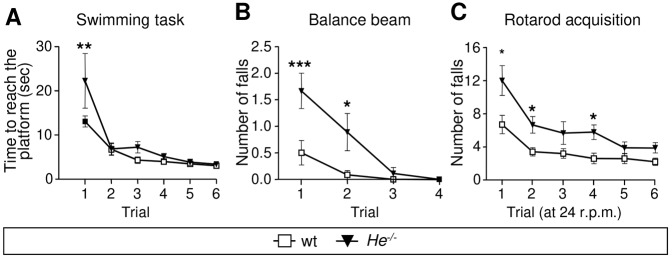
**The acquisition of new motor skills is impaired in *He^−/−^* mice.** (A-C) Motor coordination and balance were analyzed in wt and *He^−/−^* mice by performing the simple swimming test (A), the balance beam (B) and the rotarod task (C). Values are expressed as mean±s.e.m. of 7-8 mice per condition. Data were analyzed by two-way ANOVA and Bonferroni's post-hoc test. *P<0.05, **P<0.005, ***P<0.001.

To analyze the functional implication of *He* loss, we assessed the performance of motor tasks in wt and *He*^−/−^ mice (Fig. 8). In the simple swimming test, *He^−/−^* mice displayed significant abnormalities compared with wt mice in their swimming latency in the first testing trial (genotype: *F*_2,162_=4.08, *P*<0.05; post-hoc trial 1: *P*<0.01), but these disappeared over subsequent trials (Fig. 8A).

In addition, wt and *He^−/−^* mice progressively improved their performance in the balance beam along four trials (trial: *F*_3,112_=14.66, *P*<0.001). However, *He^−/−^* mice fell off more times than controls during the first trials (genotype: *F*_2,112_=13.52, *P*<0.01; post-hoc trial 1: *P*<0.001; post-hoc trial 2: *P*<0.01; Fig. 8B).

In the rotarod test, all mice reached a stable level of performance within six trials (Fig. 8C), as measured by a decrease in the number of falls in 60 s per mouse (testing trial *F*_5,138_=15.87, *P*<0.01). However, acquisition on the rotarod task was significantly delayed in *He^−/−^* compared with wt mice (genotype *F*_2,138_=21.03, *P*<0.01).

## DISCUSSION

Striatal MSNs are generated from NPCs located at the GZ of the LGE. Here, we show that He regulates late striatal neurogenesis that gives rise to D2R^+^ ENK neurons. He is expressed by NPCs in the G_1_/G_0_ cell cycle phase at the GZ, impairing the G_1_-S transition by the regulation of cyclin E, which in turn induces neuronal differentiation. Consequently, lack of *He* produces an extended S phase and cell cycle length that increases the number of proliferating NPCs at the GZ. At the beginning of the postnatal period, the number of these NPCs is reduced due to their late aberrant neurogenesis that results in cell death. These abnormalities of embryonic development in *He*^−/−^ mice produce a reduction of a specific subset of striatopallidal neurons of the dorsomedial striatum that control motor skill learning.

### *He* is necessary for striatopallidal neurogenesis

NPCs located at the GZ of the LGE become postmitotic and migrate into the MZ to acquire the MSN phenotype (Brazel et al., 2003). We have previously proposed a model for the development of striatal subpopulations in which Ikaros and He are involved in the development of striatopallidal ENK^+^ matrix MSNs (Martín-Ibáñez et al., 2012). This hypothesis is reinforced by the localization of *He* in ENK^+^ neurons that co-express D2R (present results). Besides the apparent similar function between He and Ikaros on ENK^+^ neurogenesis, there is much evidence that they determine different ENK^+^ subpopulations. They are expressed by different cells (Martín-Ibáñez et al., 2012), and their expression is not modified in the reciprocal knockout mice (Martín-Ibáñez et al., 2010, 2012). These results are contrary to the role of Ikaros family members in the hematopoietic system where they directly interact (Hahm et al., 1998; John et al., 2009), suggesting specific mechanisms of action in each system.

### He regulates neurogenesis through the control of the G1-S phase checkpoint

*Gsx2*^+^ radial glial cells constitute the first NPCs that appear during LGE ontogeny, which differentiate with the onset of the neurogenesis from the neuroephithelial cells (for a review, see Dimou and Götz, 2014). *He-*expressing cells are derived from radial glial cells, as its expression disappears in *Gsx2* knockout mice (Martín-Ibáñez et al., 2012). However, *He* loss does not compromise the number of the radial glial cell subtypes described elsewhere (Pilz et al., 2013). Radial glial cells generate the large MSNs output by a series of intermediate NPCs to amplify specific lineages, although these striatal NPCs are still poorly characterized. *He* is expressed by a small number of NPCs distributed in deep SVZ. Although the localization of *He* is mainly at the dorsal areas, it does not seem to be defining a specific SVZ domain as it has been described for other transcription factors in the VZ (Flames et al., 2007).

Some of the NPCs that express *He* at the GZ co-express low levels of Ki67. Considering that Ki67 labels cells during all phases of the cell cycle except G_0_ (Kanthan et al., 2010; Scholzen and Gerdes, 2000) and that G_1_ is the cell cycle phase with lower Ki67 expression levels (Lopez et al., 1991), we hypothesized that *He* is expressed in a subset of NPCs during G_1_ and G_0_ phases. The lack of colocalization between *He* and BrdU or PH3 reinforces the idea that *He* is not expressed by cells at S or M phases, respectively. Within G_1_ phase He impairs S-phase entry, reducing S-phase length and arresting NPCs at G_1_/G_0_ phase to facilitate neuronal differentiation. Consequently, *He*^−/−^ mice NPCs increase S-phase entry and continue proliferating in the striatal GZ impairing neurogenesis (see Fig. S18 for a representative scheme). Similarly, Lacomme and co-workers demonstrated that Ngn2 regulates G_1_-S phase transition, blocking S-phase entry and increasing the number of NPCs at G_1_/G_0_ phase (Lacomme et al., 2012). In addition, NPCs shorten S phase on commitment to neuron production (Arai et al., 2011; Turrero García et al., 2015). Thus, cell cycle length and G_1_-S phase transition are crucial processes for neurogenesis and both are regulated by He. We hypothesize that He arrests LGE-derived NPCs into phases G_1_/G_0_ to allow the accumulation of the protein machinery necessary for their differentiation to specific striatal neurons. In fact, crucial aspects of neural commitment are acquired in the final division cycle of NPCs. For example, the cortical laminar fate of NPC is acquired during the final progenitor cell division (Bohner et al., 1997; Edlund and Jessell, 1999; McConnell and Kaznowski, 1991). Similarly, during motor neuron development, NPCs become sonic hedgehog (Shh) dependent late in their final progenitor cell cycle (Ericson et al., 1996), which commits them to a motor neuronal fate (Tanabe et al., 1998).

G_1_-S phase transition is regulated by Cdk2 and cyclin E, which form a complex that participates in G_1_-S phase checkpoint (reviewed by Hardwick and Philpott, 2014; Ohtsubo and Roberts, 1993). Our results suggest that cyclin E is a key factor regulated by He that correlates with the G_1_-S phase transition impairment observed in the *He^−/−^* mice. In fact, the cyclin E gene (*Ccne1*) has two very strong He-binding domains (Kim et al., 2015) suggesting a direct regulation. Similar to our results, Pilaz and colleagues described that overexpression of cyclin E in cortical NPCs promotes a proliferation increase whereas downregulation of cyclin E led to a decrease in progenitor proliferation (Pilaz et al., 2009). In addition, a direct correlation between cyclin E and S-phase entry was proposed by ectopic expression of cyclin E, which shortens the G_1_ interval and increases the length of S phase by advancing G_1_-S phase transition (Resnitzky et al., 1994). Furthermore, ectopic expression of cyclin E can drive G_1_ cells into S phase under conditions in which Rb is not phosphorylated and E2F is not activated (Leng et al., 1997; Lukas et al., 1997). This is in agreement with our results, as we observed an increase in cyclin E but no alterations in phosphorylated RB or E2F in *He^−/−^* mice.

### *He* loss increases postnatal cell death

The homeostasis of NPCs in the striatum is a regulated process in which neurogenesis precedes astro-gliogenesis during development (Alvarez-Buylla et al., 2001; Ninkovic and Götz, 2013). However, contrary to the increase of astro-gliogenesis observed in *Ikaros^−/−^* mice (Martín-Ibáñez et al., 2010), we could not detect any effects on glial cells in *He^−/−^* mice. The role of He in neurogenesis through cyclin E-mediated G1-S transition without modifying astro-gliogenesis coincides with the effect of deferoxamine, a G_1_/S-phase blocker, which increases neuronal but not astrocytic NPC differentiation (Kim et al., 2006; Misumi et al., 2008).

The reduction of NPCs in *He^−/−^* mice at postnatal stages can be related to the increase in cell death during this period. Naturally occurring cell death is a crucial step in re-defining the final size of specific neuronal populations (Burek and Oppenheim, 1996; Kristiansen and Ham, 2014), which directly correlates with the time of prior exit from cell cycle and position during neuronal development (Gould et al., 1999). Our results point to the idea that the cell death observed in *He*^−/−^ mice is a consequence of the delay in NPCs exiting cell cycle around E18.5, then migrating into the MZ where they become neurons and die. Therefore, lack of He produces a dysfunction in the time and position of late-generated neurons in the MZ. Dual effects have also been described for Isl1 and Ebf1, which promote differentiation of striatonigral neurons and in their absence striatal cell death is observed (Garel et al., 1999; Lu et al., 2014). Taken together, all these results indicate that He loss causes aberrant neurogenesis, which in turn induces neuronal cell death compromising striatal development.

### He participates in the differentiation of a subset of MSNs that is involved in early motor learning

He-mediated regulation of the NPC cell cycle correlates with the determination of a subset of striatopallidal MSNs. The events occurring during striatal development of *He^−/−^* mice cause a specific reduction of striatal MSNs in the DMS in the adulthood. Taken together, our present findings demonstrate that He plays a direct role in the commitment of NPCs to MSNs. Accordingly, *He* overexpression is sufficient to differentiate NPCs transplanted into the striatum in MSNs expressing DARPP-32.

Previously published works and reviews suggest that striatal motor function is involved with habit formation (Yin and Knowlton, 2006) and procedural learning (Kreitzer, 2009), which fits with what we see in our *He^−/−^* mice. The striatum has been classically divided into dorsal and ventral areas, the dorsal being the most involved in motor behavior (Durieux et al., 2012). Accumulating evidence shows anatomical and functional differences in the striatum between the external DLS and the internal DMS (Durieux et al., 2012; Graybiel, 2008; Voorn et al., 2004). Interestingly, the DMS is involved in the initial stages of motor skill learning (Jueptner and Weiller, 1998; Luft and Buitrago, 2005), whereas the DLS is required for progressive skill automatization and habit learning (Miyachi et al., 2002; Yin et al., 2004). In addition, it has been shown that the loss of D2R^+^ neurons in the DMS produces early motor learning impairment but the animals can improve their performances to reach control levels (Durieux et al., 2012). As *He^−/−^* mice show impairments in the acquisition of motor skills, it seems plausible that He is involved in the generation of a specific subpopulation of striatopallidal D2R^+^ MSNs in the DMS. The cerebellum is also involved in fine-tuning the motor agility found in procedural skills. Cerebellar lesions or dysfunctions produce permanent deficits in motor tasks. However, diseased animals never perform motor tasks as well as their control or wt littermates (Sausbier et al., 2004; Stroobants et al., 2013; Vinueza Veloz et al., 2012). As *He^−/−^* mice show problems in the acquisition but not the execution of motor skills it seems that an association with cerebellar deficits is not likely.

### Conclusion

In conclusion, our results demonstrate a novel mechanism for He in the development of striatopallidal MSNs of the DMS that controls motor skills learning. He exerts its main effects on the commitment of NPCs to MSNs through the regulation of the G_1_-S phase transition and arrests NPCs at G_1_ phase to induce neuronal differentiation. The alterations of this mechanism observed in *He^−/−^* mice produce aberrant neurogenesis leading to the cell death of late-generated neurons.

## MATERIALS AND METHODS

### Animals

B6CBA wild-type (wt) mice (from Charles River Laboratories, Les Oncins, France), *He* knockout mice (*He*^−/−^) (Cai et al., 2009), pCAGs-eGFP (Okabe et al., 1997), D1R-eGFP and D2R-eGFP generated by GENSAT (Gong et al., 2003) were used. For further details of mice strains and genotyping, see the supplementary Materials and Methods.

### Birthdating, proliferation and tracking experiments *in vivo*

Birthdating experiments were performed as described elsewhere (Fig. 1B; Martín-Ibáñez et al., 2010). To study the generation of He^+^ cells, injections of EdU (50 mg/kg) at E13.5 or E14.5, or BrdU at E16.5 into wt pregnant mice were performed and allowed to develop until E18.5, when embryos were processed for He and BrdU immunohistochemistry or EdU detection (Life Technologies) (Fig. S4A).

To analyze *in vivo* proliferation in the GZ, E14.5 pregnant mice received a single dose of EdU (50 mg/kg). The proliferation analysis of E16.5, P3 and P7 was performed by Ki67 immunohistochemistry.

In order to track the origin of dead cells in the MZ, a pulse of EdU (50 mg/kg) was performed at E18.5, and immunohistochemistry was performed at P3 against EdU and cleaved caspase 3 (Cell Signaling Technology), nestin, GFAP or NeuN (Fig. 5G).

To study whether the lack of *He* could alter the cells entering the S phase of the cell cycle, we performed *in vivo* experiments with *He*^−/−^ and wt mice as previously described (Lange et al., 2009) (Fig. 4K).

For further details of these methods, see the supplementary Materials and Methods.

### Production of viral particles and cell transduction

To overexpress *He*, NPCs were transduced with the pLV-*HE*-IRES-eGFP plasmid or the pLV-IRES-eGFP plasmid, which encode human *HE* and eGFP or eGFP alone, respectively. For further details of viral particle production, see the supplementary Materials and Methods.

### Neurosphere assay

LGEs from E14.5 wt or *He^−/−^* mice were dissected out and mechanically disaggregated to culture as neurosphere and differentiate to neural cells as described previously (Martín-Ibáñez et al., 2010). For further details of neurosphere cultures, see the supplementary Materials and Methods.

Loss-of-function (LOF) experiments were performed with neurospheres derived from *He^−/−^* mice whereas gain-of-function (GOF) experiments were performed by overexpressing *He*. The number of neurons (β-III-tubulin^+^) and astrocytes (GFAP^+^) were analyzed after 6 days of differentiation.

### Cell cycle analysis *in vitro*

#### Proliferation assays

BrdU incorporation assays were performed in wt and *He^−/−^* mice-derived neurospheres (LOF) and neurospheres overexpressing *He* (GOF) as described elsewhere (Urbán et al., 2010). The number of Ki67^+^ cells was also analyzed in wt and *He^−/−^* mice-derived neurospheres (LOF) and neurospheres overexpressing *He* (GOF).

#### Cell cycle length

An accumulative exposure to 1 µM BrdU over 36 h was performed in wt and *He*^−/−^ mice-derived neurospheres (LOF) and in neurospheres overexpressing *He* (GOF) after 2 DIV in proliferation. Cells were fixed at different time points after 1 µM BrdU exposure (1, 3, 6, 12, 24 and 36 h) and processed for BrdU immunocytochemistry. Following regression analysis as previously described by Takahashi et al. (1992, 1995), the length of the cell cycle and the length of the S phase were calculated for the NPCs.

#### S-phase analysis

To study the cells entering and exiting the S phase of the cell cycle, we performed *in vitro* experiments with neurospheres derived from *He*^−/−^ and wt mice as described previously (Lange et al., 2009) (Fig. 3E-H).

#### G_2_/M phase labeling

To study the combined length of the G_2_/M phases, an accumulative exposure to 1 µM BrdU over 5 h was performed after 2 DIV in proliferation to analyze the mitotic BrdU labeling index as described previously (Takahashi et al., 1995).

#### Cell cycle index

We analyzed cell cycle index as the number of cells that incorporate BrdU but leave the cell cycle (i.e. abandoned the G_1_-S-G_2_/M phases and entered into G_0_) as previously described (Urbán et al., 2010) (Fig. S13).

#### Discerning high and low Ki67-expressing cells

We detected cells expressing high and low levels of Ki67 using the automatic intensity detection of the Cell Profiler software.

For further details of cell cycle analyses, see the supplementary Materials and Methods.

### Analysis of He-binding sites at the *Ccne1* promoter

We obtained and analyzed the Big Wig file deposited in Gene Expression Omnibus by Kim et al. (2015), and visualized it in the Integrative Genome Viewer with the files provided aligned to the Ensembl Mouse Genome. Details of database used can be found in the supplementary Materials and Methods.

### Western blots

We performed western blot analyses for cyclin E and PCNA as described elsewhere (Canals et al., 2004) in wt and *He*^−/−^ mice-derived neurospheres (LOF) and neurospheres overexpressing *He* (GOF). E2F1 and retinoblastoma (Rb) were detected in LOF experiments. For further details of western blot procedure, see the supplementary Materials and Methods.

### *In situ* hybridization

To assess which striatal subpopulation of MSNs express *He*, we performed double *in situ* hybridization for ENK or tachykinin A (*Tac1*, a precursor of SP), the precursor of SP, and immunohistochemistry for He as described previously (Martín-Ibáñez et al., 2010). For further details of *in situ* procedures, see the supplementary Materials and Methods.

### Immunolabeling

For histological preparations, embryonic or postnatal brains were removed at specific developmental stages and were frozen in dry ice-cooled methylbutane or cryoprotected depending on the procedure. Immunolabeling was performed according to the protocols described by Bosch et al. (2004) and Canals et al. (2004). For further details of the antibodies used and immunostaining procedures, see the supplementary Materials and Methods.

### Measurement of volumes and *in vivo* cell counts

The volumes of certain brain regions were measured using ImageJ v1.33 as described previously (Canals et al., 2004). All cell counts [EdU and Ki67 for GZ proliferation; BrdU for birthdating experiments; cleaved caspase 3 for cell death; *Ctip2*, calbindin, DARPP-32, choline acetyl transferase (ChAT) and parvalbumin for striatal cell population] were performed blind to genotype. Unbiased stereological counts were performed for all striatal areas for each animal. DMS, DLS, VMS and VLS were specifically counted for DARPP-32-, ENK- and SP-positive cells.

The distribution of mitosis in *He^−/−^* and wt striatum at E16.5 was analyzed as described by Pilz et al. (2013) and counted using CAST software.

Automated quantification of branches, and neurite length was performed using Cell Profiler v2.8 software.

For further details of cell counts, see the supplementary Materials and Methods.

### Q-PCR

Gene expression was evaluated by Q-PCR assays as previously described by Martín-Ibáñez et al. (2010). For further details of the probes used and PCR procedures, see the supplementary Materials and Methods.

### Primary striatal culture and transfection

E14.5 fetal LGEs were dissected and cultured as previously described (Martín-Ibáñez et al., 2010). For *He* overexpression studies, cells were transfected with the MSCV-*He*-IRES-eGFP plasmid, or with the MSCV-IRES-eGFP plasmid as a control (Zhang et al., 2007). We counted the number of *He* or eGFP overexpressing cells that colocalized with calbindin, DARPP-32 or ENK. For further details of primary culture methods, see the supplementary Materials and Methods.

### Cell transplants

Unilateral striatal injections of *He*-overexpressing cells were performed using a stereotaxic apparatus (Davis Kopf Instruments, Tujunga, CA, USA); coordinates (mm): AP, +2.3, L, +1.4 from lambda and DV, −1.8 from dura. For further details of cell transplants, see the supplementary Materials and Methods.

### Mouse behavior

#### Swimming task

The mice were placed at the end of a transparent perspex extended swimming tank facing away from a visible escape platform at one end of the tank and the time taken to reach the platform was recorded.

#### Balance beam

Animals were allowed to walk along a horizontally placed beam of a long steel cylinder (50 cm) with 15 mm diameter. Latency to fall and number of falls were measured.

#### Rotarod

Acquisition of a motor coordination task was further evaluated on the rotarod apparatus (24 rpm). Latency to fall and the number of falls during 60 s was recorded.

For further details of mouse behavior analyses, see the supplementary Materials and Methods.

### Statistical analysis

All results are expressed as the mean of independent experiments±s.e.m. Results were analyzed using Student's *t*-test or one-way or two-way ANOVA, followed by the Bonferroni post-hoc test.
